# Design of Experiment (DOE) Utilization to Develop a Simple and Robust Reversed-Phase HPLC Technique for Related Substances’ Estimation of Omeprazole Formulations

**DOI:** 10.3797/scipharm.1306-06

**Published:** 2013-08-12

**Authors:** Vayeda Chintan Manranjan, Devendra Singh Yadav, Hitesh Amrutlal Jogia, Praful Lalitkumar Chauhan

**Affiliations:** Analytical Research and Development, Integrated Product Development, Dr. Reddy’s Laboratories Ltd., Bachupally, Hyderabad-500 072, India.

**Keywords:** DOE, HPLC/UPLC, Compatible, Related Substances, Anova, Method development, Chromatography

## Abstract

A simple, fast, and sensitive reversed-phase HPLC method with UV detection was developed for the quantitation of omeprazole and its eleven related compounds (impurities) in pharmaceutical formulation using the Thermo Accucore C–18 (50 mm × 4.6 mm, 2.6 μm) column. The separation among all the compounds was achieved with a flow rate of 0.8 mL min^−1^ employing a gradient program of mobile phase A [0.08 M glycine buffer pH 9.0: acetonitrile; 95:05 (v/v)] and mobile phase B [acetonitrile: methanol; 65:35 (v/v)]. The chromatographic detection was carried out at a wavelength of 305 nm. The method was validated for specificity, linearity, and recovery. The huskiness of the method was determined prior to validation using the Design of Experiments (DOE). The ANOVA analysis of DOE with a 95% confidence interval (CI) confirmed the buffer pH of mobile phase A (p <0.0001) and column temperature (p<0.0001) as significant Critical Method Parameters (CMPs).

## Introduction

Over the last few years, there seems to have been a marked strengthening of the requirements for the isolation and separation of impurities resulting from active pharmaceutical ingredients by regulatory agencies [1]. As a consequence, the analytical technology for impurity analysis has improved beyond the traditional chromatographic and spectroscopic methods [2].

Omeprazole, 5-methoxy-2-{[(4-methoxy-3,5-dimethylpyridin-2-yl)methyl]sulfinyl}-1*H*-benz-imidazole, is one of the most frequently prescribed drugs in patients with gastro-esophageal reflux disease (GERD) [3]. Pharmacopoeial monographs and literature are available for the stability-indicating HPLC and UPLC method for omeprazole/esomeprazole, in which separation was achieved among the drug and a maximum of around seven related compounds (impurities) [4–8].

**Fig. 1:**
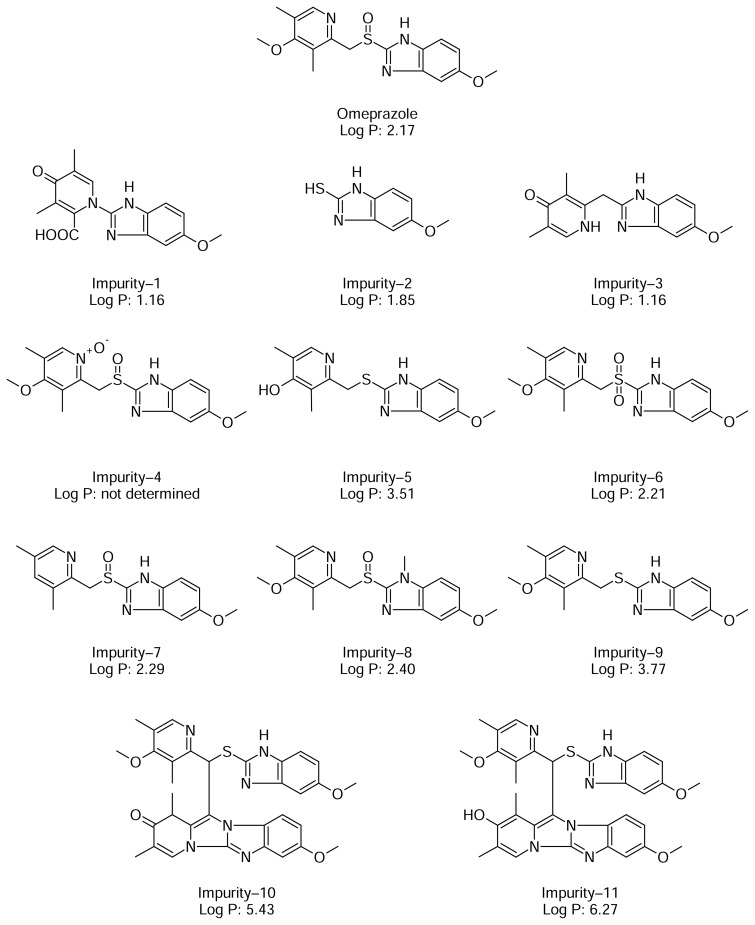
Structure of omeprazole and all related compounds with Log P values

However, omeprazole has more than seven related impurities (including pharmacopoeial/non-pharmacopoeial) which needs to be separated in a chromatographic run (Fig. 1). Moreover, some laboratories which are governed by respective national regulatory agencies do not encourage the use of UPLC as it is not commonly available. Thus, to subdue this issue, the idea was to develop an HPLC/UPLC compatible method, which is a formidable task.

The method was developed using the one-factor-at-a-time (OFAT) approach, where one variable changes sequentially until a suitable method is shaped, which is an old approach. This type of method development provides limited understanding of a method’s capabilities and robustness. A quality-by-design approach uses statistical Design of Experiments to develop a rugged method with ‘design space’. The design space defines the experimental region in which changes to method parameters will not significantly affect the quality and results of the method. This approach strengthens the robustness of the method being developed [9, 10]. Sabrina Flor et al. exploited the experimental design concept to assess the ruggedness of the stability-indicating method for omeprazole, only considering the closely eluting impurity to the main peak [11].

In this work we adopted the QbD principle to develop the HPLC/UPLC-compatible stability-indicating method for omeprazole’s Dual Delayed Release (DDR) to attain the desired separation. The Accucore column packed with pellicular particles was explored to achieve the desired separation among omeprazole and its related impurities with the reduced run time.

## Experimental

### Chemicals and Reagents

All standards and test samples were supplied by Dr. Reddy’s Laboratories Limited, Hyderabad, India; i.e. omeprazole, Impurity-1 (1-(5-methoxy-1*H*-benzimidazol-2-yl)-3,5-dimethyl-4-oxo-1,4-dihydropyridine-2-carboxylic acid), Impurity-2 (5-methoxy-1H-benz-imidazole-2-thiol), Impurity-3 (2-[(5-methoxy-1*H*-benzimidazol-2-yl)methyl]-3,5-dimethyl-pyridin-4(1*H*)-one), Impurity-4 (5-methoxy-2-{[(4-methoxy-3,5-dimethyl-1-oxidopyridin-2-yl)methyl]sulfinyl}-1*H*-benzimidazole), Impurity-5 (2-{[(5-methoxy-1*H*-benzimidazol-2-yl)-sulfanyl]methyl}-3,5-dimethylpyridin-4-ol), Impurity-6 (5-methoxy-2-{[(4-methoxy-3,5-di methylpyridin-2-yl)methyl]sulfonyl}-1*H*-benzimidazole), Impurity-7 (2-{[(3,5-dimethylpyridin-2-yl)methyl]sulfinyl}-5-methoxy-1*H*-benzimidazole), Impurity-8 (5-methoxy-2-{[(4-methoxy-3,5-dimethylpyridin-2-yl)methyl]sulfinyl}-1-methyl-1*H*-benzimidazole), Impurity-9 (5-meth-oxy-2-{[(4-methoxy-3,5-dimethylpyridin-2-yl)methyl]sulfanyl}-1*H*-benzimidazole), Impurity-10 (8-methoxy-12-{[(5-methoxy-1*H*-benzimidazol-2-yl)sulfanyl](4-methoxy-3,5-dimethylpyr-idin-2-yl)methyl}-1,3-dimethylpyrido[1′,2′:3,4]imidazo[1,2-*a*]benzimidazol-2(1*H*)-one), Impu-rity-11 (8-methoxy-12-{[(5-methoxy-1*H*-benzimidazol-2-yl)sulfanyl](4-methoxy-3,5-dimeth-ylpyridin-2-yl)methyl}-1,3-dimethylpyrido[1′,2′:3,4]imidazo[1,2-*a*]benzimidazol-2-ol). HPLC grade acetonitrile, methanol, and analytical grade glycine, borax, and phosphoric acid were procured from Merck, Darmstadt, Germany. Water was obtained by using the Millipore Milli-Q Plus water purification system.

### Equipment

The HPLC (Agilent, USA) used consisted of a quaternary solvent manager, a sample manager, and a UV-Visible multiple wavelength detector. The output signal was monitored and processed using Empower-2 software.

The UPLC (Waters, USA) used consisted of a quaternary solvent manager, a sample manager, and a photodiode array wavelength detector. The output signal was monitored and processed using Empower 2 software.

### Briefly about the Formulation

The omeprazole DDR formulation is a mixture of delayed (DR) and extended (ER) release pellets [12], where the solubility and dispersion of the DR part is pH-dependent; while the ER part is pH-independent. The DR part of the formulation is likely to facilitate a loading dose of the drug, while the ER part is prone to maintain the concentration of the drug.

### Chromatographic Conditions

The Thermo Scientific Accucore C-18 (50 mm × 4.6 mm, 2.6 μm) was used as the chromatographic column. The separations among all the compounds were achieved with a flow rate of 0.8 mL min^−1^ using a gradient program of mobile phase A and mobile phase B with a 5 μL injection volume. Where mobile phase A consisted of a 0.08 M glycine (pH 9.0) buffer and acetonitrile in a 95:05 (v/v) ratio, mobile phase B was prepared by mixing acetonitrile and methanol in the ratio of 65:35 (v/v), respectively. The HPLC gradient program was set as: time (min) vs. % mobile phase A: 0/100, 1/100, 11.8/92, 23.3/75, 30.5/60, 37.7/30, 43/30, 45/100, and 50/100. The column temperature was maintained at 30°C and the detection was monitored at 305 nm wavelength. A mixture of 0.01 M borax and ethanol in the proportion of 80:20 (v/v) was used as a solvent/diluent.

### Preparation of Standard Solution

A stock solution of omeprazole standard (60 μg mL^−1^) was prepared by dissolving an accurately weighed amount of omeprazole standard in diluent. The final concentration of the standard was kept at 1.8 μg mL^−1^ by diluting an appropriate volume of stock solution with diluent.

### Preparation of System Suitability Solution

A suitable amount of omeprazole and nine other impurities (Impurity–1 to Impurity–9) were dissolved in diluent to prepare a mixture that contained 600 μg mL^−1^ of omeprazole and 1.8 μg mL^−1^ of each impurity.

### Preparation of the Test

Omeprazole pellets equivalent to 600 mg of omeprazole were dissolved in diluent by sonication for 30 minutes with intermittent shaking. The final concentration of the test was maintained at 600 μg mL^−1^ using diluent. The sample solution was filtered through a 0.45μm PVDF hydrophilic membrane filter. Likewise, one more sample was prepared with omeprazole pellets and diluent, in which all nine impurities (Impurity–1 to Impurity–9) were spiked at 0.5% of the analyte concentration.

### Experimental Design

Utilizing the two-level full factorial design options, the experimental design was created in Design Expert V8 software for Critical Method Parameters (CMPs). Evaluation of the selected (Tab. 3) Critical Quality Attributes (CQAs) was carried out by ANOVA statistics for experimental design.

## Results and Discussion

### Method Optimization

During the stressed and stability studies it was found that the in–house developed omeprazole formulation generated eleven impurities, out of which five impurities (Impurities–2, 4, 6, 7, and 9) were pharmacopoeial, while the other six impurities (Impurities–1, 3, 5, 8, 10, and 11) were non-pharmacopoeial (Fig. 1) [13, 14]. Structure elucidation and characterization were carried out in-house for six non–pharmacopoeial impurities (data not published).

The purpose of the work was to develop a HPLC/UPLC-compatible method capable of separating omeprazole from its eleven related impurities in a single chromatographic run. The structure and Log P values (obtained using ChemDraw V12) of omeprazole and its impurities (Fig. 1) indicate that all compounds have similar functional groups, but are dissimilar in polarity. Therefore, a buffer salt capable of reacting reciprocally is required for buffer preparation. The amphoteric characteristics of the buffer salt, glycine, which increases the durability of the silica-based reversed-phase column [15], was selected for the buffer preparation of mobile phase A. The pH of the mobile phase A buffer was selected based on the stability of omeprazole in basic solution [16]. The desired separation of all impurities with the HPLC method using a traditional C18 column (100 mm × 4.6 mm, 5 μm) was achieved within 120 min, hence, the target was to develop a compatible method with a shorter run time. Development was initiated using only small length columns i.e. Waters: X-Bridge C18 Column (50 mm × 4.6 mm, 5 μm) with a flow rate that varies from 1.0 mL min^−1^ to 0.8 mL min^−1^ using a gradient program of mobile phase A and mobile phase B. The perceptible separation was not achieved using a Waters X-Bridge column among the drug peak and related impurities. Consequently, the stationary phase was replaced with a column packed with pellicular particles (Agilent Poroshell), which are not totally porous particles, but rather they have a solid core and a thin porous outer layer. The thin porous layer of these particles allows very rapid access of molecules to interactive surfaces within the porous structure. The result is that high mobile phase velocity can be used for very fast separations with good column efficiency [17–19]. Eventually, the first satisfactory chromatographic separation was achieved among omeprazole and its related compounds using the Agilent Poroshell 120 EC–C18 (50 mm × 4.6 mm, 2.7 μm) column with a flow rate of 0.8 mL min^−1^ applying a gradient program of mobile phase A; 0.08 M glycine (pH 9.0) buffer: acetonitrile 95:05 (v/v) and mobile phase B; acetonitrile: methanol 65:35 (v/v), respectively. However, the Agilent column only has a viable pH range of 2–9 [18]. Because of this, the column was substituted by the Thermo Accucore C–18 (50 mm × 4.6 mm, 2.6 μm) column, packed with similar pellicular stationary phases and additionally, has a higher pH working range (i.e. pH 2–11) [19]. Separation among all compounds was achieved by using a gradient program with a slight modification of mobile phase A with respect to time i.e. min/% of mobile phase A: 0/100, 1/100, 11.8/92, 23.3/75, 30.5/60, 37.7/30, 43/30, 45/100, and 50/100.

Omeprazole and its related impurities were well-separated in the optimized chromatographic conditions {Fig. 2 (A), (B), (C), and (D)} on the HPLC and UPLC. The retention times of all impurities was confirmed by injecting individual impurity solutions. Impurity-10 and Impurity-11 were removed from the system suitability due to the unavailability of the impurities’ standards.

Likewise, the relative response factor (RRF) and linearity were determined for the eight impurities instead of the eleven impurities due to the unavailability of the Impurity-3 standard in sufficient quantity.

### Specificity

The specificity of the chromatographic method was verified using a placebo (all formulation substances excluding the active one) instead of the actual formulation. No interference was observed either at the retention time of the impurities or at the retention time of omeprazole. This indicates that the method is specific and selective for omeprazole and its related compounds’ analysis {Fig. 2(B)}.

### Linearity and Recovery

The results of retention time, relative retention time, and resolution from the adjacent peak, peak tailing, and RRFs are captured in Tab. 1. Linearity solutions for the related compounds and omeprazole were prepared by serial dilution of the impurity stock solutions and the omeprazole stock solution. Five different concentrations between 0.6 μg mL^−1^ (0.1% of the test preparation) and 6.0 μg mL^−1^ (1.0% of test preparation) were prepared for linearity. The maximum permissible limit for all omeprazole-related compounds is 0.3% (based on the dose value) for an analyte concentration of 600 μg mL^−1^.

The correlation coefficients, slopes, and y-intercepts of the calibration plots are reported (Tab. 2) for the eight related compounds. The correlation coefficient values are >0.998 for all the components and the y-intercept bias is within ±2.0% of 100% linearity response (Tab. 2). These results indicate that the method is linear up to the specified range of concentrations. The accuracy study of related compounds was performed at 0.3% and 0.6% levels of the analyte concentration (600 μg mL^−1^) (Tab. 2).

### Experimental Design

The ability of a chromatographic method to successfully separate, identify, and quantitate species was determined by a number of factors, many of which are in the control of the experimenter. Attempting to discover the importance of these factors with respect to the response, the experimental design/Design of Experiments (DOE) gives a powerful suite of statistical methodology, which is capable of estimating the effects of each factor in combination as well as alone. Based on the experience of development, flow rate, pH of the glycine buffer used to prepare mobile phase A, % of acetonitrile in mobile phase B, and the column temperature were identified as Critical Method Parameters (CMPs) for DOE to evaluate the huskiness of the method (Tab. 3).

The resolutions between Impurity-1 and Impurity-2 (Response–1) as well between Impurity-5 and Impurity-6 (Response–2) were found to be critical, as they get affected by selected CMPs. Henceforth, these two responses were selected as Critical Quality Attributes (CQAs) to assess the effect of CMPs (Tab. 3). Two-level full-factorial DOE, with three centre points, were generated using Design Expert software (n=19). The ANOVA analysis with a 95% confidence interval (CI) for the chosen responses of the DOE reveals the existence of curvature, which indicates a systematic contribution of the independent factor that is not accounted by the model. Hence, the design was orthogonally augmented with two centre points and eight axial points. (Total n=29).

Again, the responses were analyzed by ANOVA with a 95% CI. The DOE results identified the column temperature and pH as the most critical parameters (p < 0.05) for the selected responses (Tab. 4). The perturbation plot {Fig. 3(A) and (C)} displays the effect of all the factors at a particular point in the design space for the selected responses.

The response was plotted by changing only one factor over its range while holding all the other factors constant. The design space {Fig. 3(B) and (D)} is the region in which changes to the method parameters will not significantly affect the results. During the experiment it was found that both of the responses are inversely proportional to each other, i.e. if Response-1 increases more than 6.5, Response-2 decreases less than 2, which was not advantageous. Hence, the criteria set to obtain the design space for Response-1 and Response-2 was the resolution between 3.2 to 6.5 and the resolution 2 to 4.5, respectively.

## Conclusion

The QbD-based reversed-phase HPLC method for the determination of impurities of omeprazole DDR was developed with a column containing a pellicular stationary phase. The method was evaluated in terms of specificity, linearity, and recovery. The CMPs were identified and applied to DOE, which displays the gruffness of the chromatographic method prior to validation. The method was found to be robust within the defined design space. This method is capable of achieving separation among omeprazole and its eleven related compounds in a short run time and is useful in the regions where regulatory agencies recommend HPLC over UPLC analytical methods.

However, the limit of detection and limit of quantification for all compounds need to be carried out in the future and the RRF establishment for Impurity-3, Impurity-10, and Impurity-11 needs to be executed further. The summary of the work advocates that this stability-indicating method is capable of achieving separation among omeprazole and its eleven related compounds in only 45 minutes.

## Figures and Tables

**Fig. 2(A):**
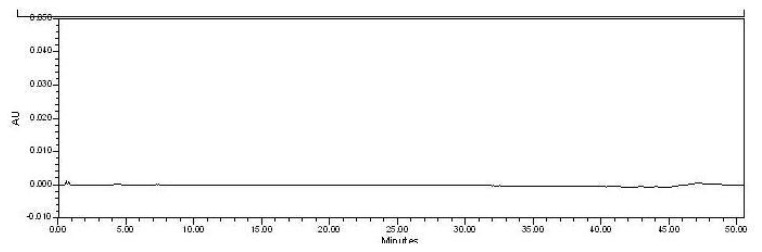
Specimen Chromatogram of Placebo injected in HPLC

**Fig. 2(B):**
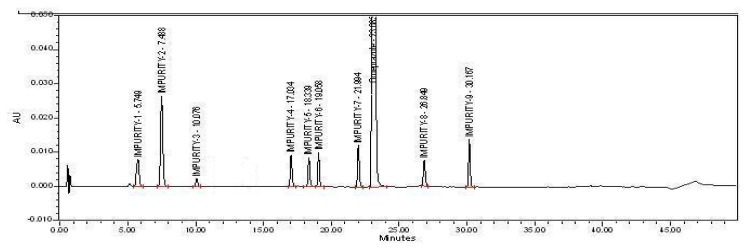
Specimen Chromatogram of System Suitability injected in HPLC

**Fig. 2(C):**
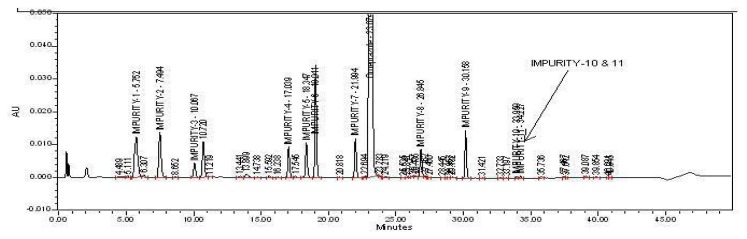
Specimen Chromatogram of Spiked Test injected in HPLC

**Fig. 2(D):**
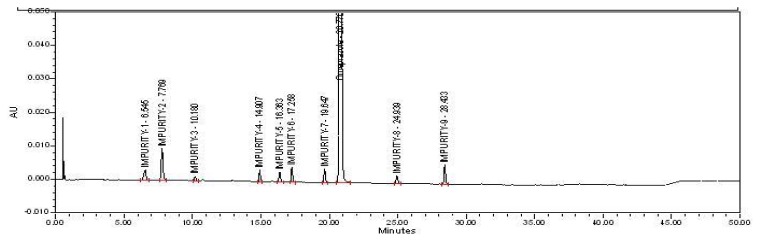
Specimen Chromatogram of System Suitability injected in UPLC

**Fig. 3(A):**
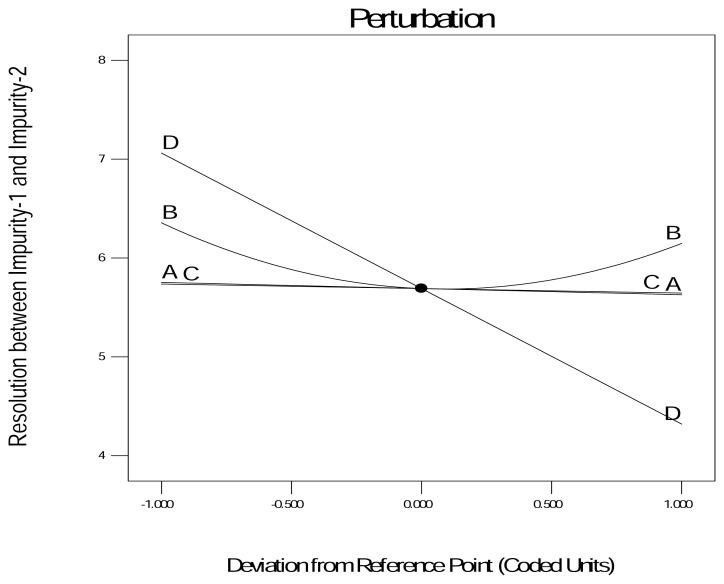
Perturbation graph for Response-1

**Fig. 3(B):**
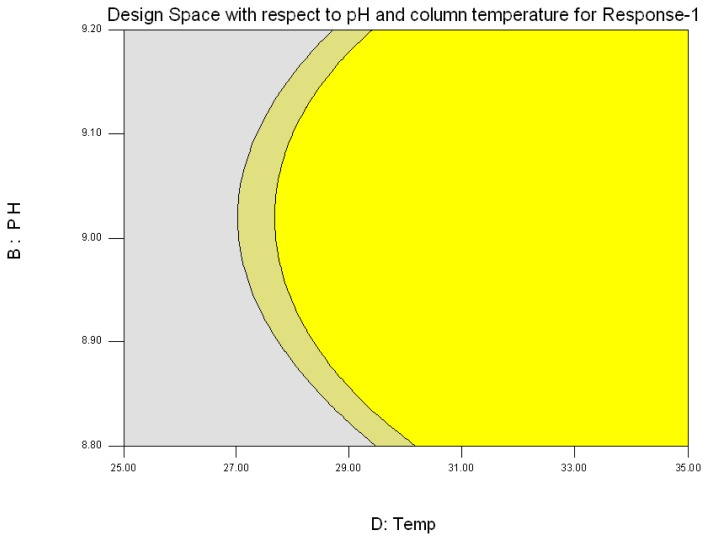
Design Space (Yellow color) for Response-1

**Fig. 3(C):**
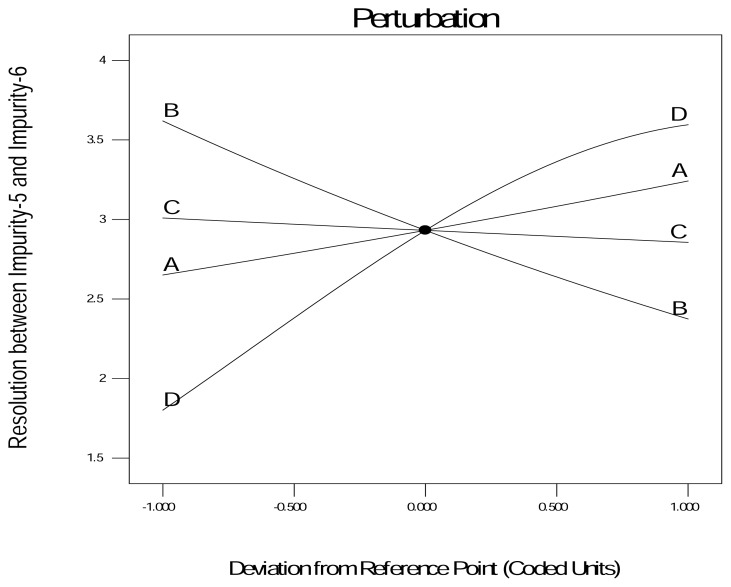
Perturbation graph for Response-2

**Fig. 3(D):**
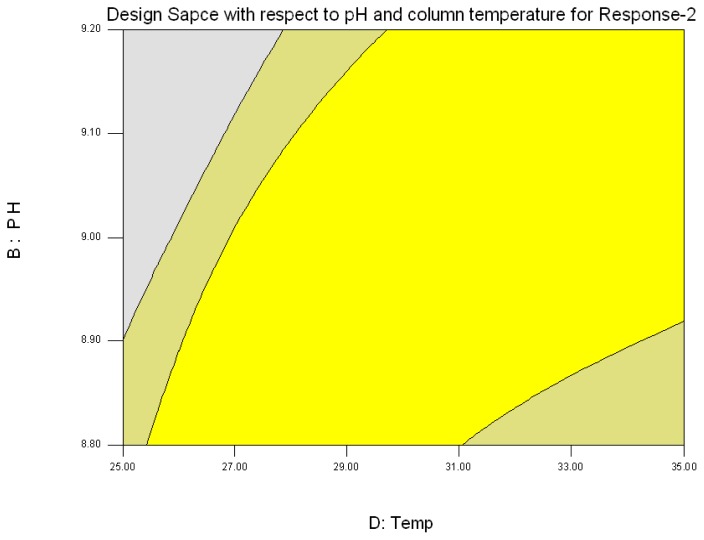
Design Space (Yellow color) for Response-2

**Tab. 1:** Retention Time, Relative Retention Time, Resolution from adjacent peak, peak tailing, and RRF for final chromatographic method.

Compound Name	Retention Time	Relative Retention Time	Resolution	Tailing Factor	RRF
Omeprazole	23.085	NA*	About 4.5	About 1.1	
Impurity-1	5.749	About 0.25	NA*	About 1.1	1.24
Impurity-2	7.490	About 0.33	About 5.3	About 1.1	3.09
Impurity-3	10.078	About 0.43	About 8.5	About 1.1	ND*
Impurity-4	17.034	About 0.74	About 26.4	About 1.1	0.94
Impurity-5	18.339	About 0.80	About 5.1	About 1.1	0.98
Impurity-6	19.058	About 0.82	About 2.8	About 1.1	1.01
Impurity-7	21.994	About 0.95	About 12.8	About 1.1	1.15
Impurity-8	26.849	About 1.16	About 14.4	About 1.1	0.82
Impurity-9	30.167	About 1.31	About 13.2	About 1.1	1.07
Impurity-10	33.921	About 1.47	About 4.5	About 1.1	ND*
Impurity-11	34.189	About 1.48	About 1.5	About 1.1	ND*

* ND: Not Determined; NA: Not Applicable.

**Tab. 2:** Linearity and recovery in final chromatographic method

Compound Name	Slope	Intercept	Correlation coefficient	Bias	Recovery at 0.3%	Recovery at 0.6%
Omeprazole	15503.0	359.59	0.9999	0.76	100%	100%
Impurity-1	19267.4	503.42	0.9999	0.88	91%	92%
Impurity-2	47909.1	1596.08	0.9998	1.09	95%	94%
Impurity-3	ND	ND	ND	ND	ND	ND
Impurity-4	14574.4	−8.46	0.9999	−0.02	95%	95%
Impurity-5	15251.5	−80.75	0.9999	−0.18	91%	91%
Impurity-6	15723.5	958.05	0.9996	1.96	97%	97%
Impurity-7	17866.7	301.95	1.0000	0.56	95%	96%
Impurity-8	12636.2	723.12	0.9999	1.84	97%	97%
Impurity-9	16614.1	289.97	1.0000	0.58	94%	95%
Impurity-10	ND*	ND*	ND*	ND*	ND*	ND*
Impurity-11	ND*	ND*	ND*	ND*	ND*	ND*

* ND: Not Determined.

**Tab. 3:** CMP, CQA, and QTMP

Omeprazole DDR Stability-Indicating Analysis Method				

CMP(Critical Method Parameter)	Range of each parameters used for DOE		QTMP(Quality Target Method Profile)	CQA (Critical Quality Attribute)

	Low	High	Targeted QTMP	
A) Flow rate	0.6 mL/min	1.0 mL/min	Resolution should be not less than 2	Resolution between Impurity-1 and Impurity-2
B) pH of glycine buffer used to prepare mobile phase-A	pH 8.6	pH 9.4		
C) % of Acetonitrile in mobile phase-B	45%	85%		Resolution between Impurity-5 and Impurity-6
D) Column Oven Temperature	20°C	40°C		

**Tab. 4:** ANOVA analysis

Parameters selected for ANOVA Analysis	Resolution between Impurity-1 and Impurity-2		Resolution between Impurity-5 and Impurity-6		Acceptance criteria
	p Value for ANOVA	Significance	p Value for ANOVA	Significance	
A-Flow	0.4630	Not significant	0.0833	Not significant	p should be less than 0.05 for Significant
B-pH	0.2262	Not significant	0.0009	Significant	
C-% of Acetonitrile	0.5893	Not significant	0.6429	Not significant	
D-Column Temperature	<0.0001	Significant	<0.0001	Significant	
AD	0.0442	Significant	Not selected	NA	
B2	<0.0001	Significant	Not selected	NA	
D2	Not selected	NA	0.0113	Significant	
Model	<0.0001	Significant	<0.0001	Significant	
Lake of Fit	0.8665	Not significant	0.5576	Not significant	
